# Autovaccination Confers Protection against *Devriesea agamarum* Associated Septicemia but Not Dermatitis in Bearded Dragons (*Pogona vitticeps*)

**DOI:** 10.1371/journal.pone.0113084

**Published:** 2014-12-05

**Authors:** Tom Hellebuyck, Katleen Van Steendam, Dieter Deforce, Mark Blooi, Filip Van Nieuwerburgh, Evelien Bullaert, Richard Ducatelle, Freddy Haesebrouck, Frank Pasmans, An Martel

**Affiliations:** 1 Department of Pathology, Bacteriology and Avian diseases, Faculty of Veterinary Medicine, Ghent University, Merelbeke, Belgium; 2 Laboratory for Pharmaceutical Biotechnology, Faculty of Pharmaceutical Science, Ghent University, Ghent, Belgium; 3 Centre for Research and Conservation, Royal Zoological Society of Antwerp, Antwerp, Belgium; Indian Institute of Science, India

## Abstract

Devrieseasis caused by *Devriesea agamarum* is a highly prevalent disease in captive desert lizards, resulting in severe dermatitis and in some cases mass mortality. In this study, we assessed the contribution of autovaccination to devrieseasis control by evaluating the capacity of 5 different formalin-inactivated *D. agamarum* vaccines to induce a humoral immune response in bearded dragons (*Pogona vitticeps*). Each vaccine contained one of the following adjuvants: CpG, incomplete Freund's, Ribi, aluminium hydroxide, or curdlan. Lizards were administrated one of the vaccines through subcutaneous injection and booster vaccination was given 3 weeks after primo-vaccination. An indirect ELISA was developed and used to monitor lizard serological responses. Localized adverse effects following subcutaneous immunization were observed in all but the Ribi adjuvanted vaccine group. Following homologous experimental challenge, the incomplete Freund's as well as the Ribi vaccine were observed to confer protection in bearded dragons against the development of *D. agamarum* associated septicemia but not against dermatitis. Subsequently, two-dimensional gelelectrophoresis followed by immunoblotting and mass spectrometry was conducted with serum obtained from 3 lizards that showed seroconversion after immunisation with the Ribi vaccine. Fructose-bisphosphate aldolase and aldo-keto reductase of *D. agamarum* reacted with serum from the latter lizards. Based on the demonstrated seroconversion and partial protection against *D. agamarum* associated disease following the use of formalin-inactivated vaccines as well as the identification of target antigens in Ribi vaccinated bearded dragons, this study provides promising information towards the development of a vaccination strategy to control devrieseasis in captive lizard collections.

## Introduction

*Devriesea agamarum* is the causative agent of chronic proliferative dermatitis and septicemia in several genera of desert-dwelling lizards [1], [2], [3]. *D. agamarum* related disease appears to be highly contagious and may affect a complete lizard collection within several months [1], [4]. While in dab lizards (*Uromastyx* species) mortality remains low despite high morbidity, considerable mortality occurs in other agamid and iguanid species [3]. Recently, *D. agamarum* was shown to be able to persist for several years in captive lizard colonies [5]. Persistence is promoted by prolonged environmental survival of the bacterium as well as the existence of asymptomatic carriers, which form a major reservoir for *D. agamarum* infection [1], [5],[6]. Successful antimicrobial treatment and efficient disinfection procedures have previously been established to control *D. agamarum* associated disease [2], [6]. Besides quarantine and entry control of newly acquired lizards [4], other preventive measures against *D. agamarum* associated disease in captive lizard collections, do not exist. Prophylactic immunization of lizards could offer a powerful tool to prevent introduction or spread of the disease into captive collections and/or to reduce the severity of infection.

Like all jawed vertebrates, reptiles have both an innate and adaptive immune system [7]. Nevertheless, immune function of reptiles has received relatively minor attention and little is known concerning the existence of affinity maturation in lizards and other reptiles [8], [9]. More than in other vertebrates, the immune response in these ectothermic amniotes is influenced by a variety of environmental as well as seemingly species dependent factors [7]. Moreover, differences in antigen properties and route of antigen uptake account for highly variable immune responses in lizards [10]. Presently, there are only two documented examples of challenge/vaccination experiments in reptiles [8], [9].

The purpose of the present study was to determine the effect of prophylactic immunization of bearded dragons (*Pogona vitticeps*) against the *D. agamarum* type strain. First, the development of a humoral immune response was assessed following the administration of 5 different formalin-inactivated *D. agamarum* vaccines in bearded dragons. Next, the most suitable vaccine formulations were selected to conduct challenge/vaccination experiments. Finally, the target antigens of the induced antibodies were identified.

## Materials and Methods

### Preparation of a formalin-killed *Devriesea agamarum* suspension and challenge inoculum

The type strain of *D. agamarum* ( = LMG 24257^T^ = IMP 2) was used to prepare bacterial suspensions for immunization, experimental inoculation and western blotting. Suspensions were prepared after incubation of *D. agamarum* on Columbia agar with 5% sheep blood (COL, Oxoid GmbH, Wesel, Germany) during 24 h at 37°C and 5% CO_2_.

For vaccine preparation, ten *D. agamarum* colonies were transferred to 100 ml of Columbia broth and incubated during 24 h at 37°C and 5% CO_2_. A 10-ml aliquot was taken from the broth, pelleted by centrifugation (3000 rpm, 10 minutes, 4°C) and suspended in phosphate buffered saline (PBS). Subsequently, the number of colony-forming units (cfu) was determined by plating serial tenfold dilutions on COL agar. The suspension had an optic density of 1.560, which equalled 10^9^ cfu/ml. Next, the broth was supplemented with 36% formalin to a final concentration of 0.5% and incubated overnight at 37°C. After centrifugation (5000 rpm, 30 minutes, room temperature), bacteria were suspended in PBS. To confirm complete killing, 50-µl aliquots of the bacterial suspension were plated onto COL agar, incubated at 37°C and 5% CO_2_ during 48 h.

To prepare the challenge inoculum, 10 colonies were harvested and incubated during 24 h in 5 ml of brain heart infusion (BHI, Merck, Darmstadt, Germany) broth at 37°C and 5% CO_2_. Following centrifugation (3000 rpm, 10 minutes, 4°C) the bacteria were washed three times in 5 ml of phosphate buffered saline (PBS). The inoculum was diluted with PBS to an optic density of 1.050, which equaled 10^8^ cfu/ml.

### ELISA for the evaluation of the antibody response against the *Devriesea agamarum* type strain

An indirect ELISA was developed to assess the antibody response in bearded dragons (*P. vitticeps*) following immunization against *D. agamarum*. All experiments were performed with the permission of the Ethical Committee of the Faculty of Veterinary Medicine, Merelbeke, Ghent University, Belgium (authorization number 2009_071 and 2009_089). During all experiments lizards were housed individually in a room where the temperature was maintained at 28°C during the day and 20°C during the night. A 12-hour photoperiod was given with a self-ballasted bulb installed above each enclosure, creating a local hot spot.

#### Rabbit sera

Anti-lizard immunoglobulin serum was prepared in rabbits after immunization with lizard immunoglobulins. Immunoglobulins were collected from lizard serum obtained from healthy, adult *P. vitticeps* using the ammonium precipitation method and subsequent dialysis, as previously described by Pasmans et al. [11]. Rabbits were immunized with 1 mg of the purified protein fraction in 1 ml of 50% incomplete Freund's adjuvant (Sigma-Aldrich, Bornem, Belgium). Subsequent immunizations were administered on days 14 and 28. Rabbits were anesthetized and exsanguinated on day 42. Plasma was separated and stored at −70°C.

#### Serological response of bearded dragons (*Pogona vitticeps*) immunized with 5 different inactivated *Devriesea agamarum* vaccines

Twenty-five clinically healthy 1.5-year-old bearded dragons, weighing 140 to 190 g, were immunized with the formalin-inactivated *D. agamarum* type strain. All products were purchased from Sigma-Aldrich, Bornem, Belgium unless stated otherwise. Five groups of 5 lizards each received one of the following vaccines, each containing a total of 1×10^8^ cfu, through subcutaneous injection at the dorsolateral skin region: 1) 100 µl of 30% CpG vaccine (CpG oligodeoxynucleotide, Biosource, Nivelles, Belgium), 2) 200 µl of 50% incomplete Freund's adjuvant vaccine, 3) 100 µl vaccine suspension emulsified in Ribi adjuvant (50 µg monophosphoryl lipid A plus 50 µg synthetic trehalose dimycolate), 4) 200 µl of 50% aluminium hydroxide vaccine, and 5) 100 µl of 30% curdlan vaccine. Preimmune heparinized blood samples were collected prior to primo-vaccination. Subsequently, blood was collected weekly during 7 weeks and booster vaccination was given after 21 days. All bearded dragons were examined daily for the development of adverse effects following immunization. Signs of generalized effects such as anorexia and apathy or localized skin alterations at the site of injection such as skin discoloration or the development of dermal inflammation, were closely monitored in all immunized lizards during a 100 days observation period.

#### ELISA procedure

Wells of 96-well microtiter plates (Maxisorp, Nunc, Roskilde, Denmark) were coated with 150 µl of a formalin-inactivated *D. agamarum* suspension of 7×10^7^ cfu/ml in 0.05 M carbonate-bicarbonate buffer (pH 9.6) and incubated for 24 h at 4°C. The plates were washed 4 times with PBS supplemented with 0.05% Tween 20 (washing buffer), dried and stored at 4°C. Between each incubation step, the wells were washed 5 times. Lizard sera were diluted 1∶64 in washing buffer with 2.2% skim milk powder. Preimmune as well as immune serum samples from individual lizards were analysed in 3-fold and incubated on the same antigen coated plate in order to minimize variability of demonstrated OD values resulting from differences in coating and further processing of the plates. One-hundred microliters of diluted lizard serum samples were added to each well and the plates were incubated for 2 h at 37°C. Subsequently, the wells were incubated with 100 µl of rabbit anti-lizard serum, diluted 1∶7000 in washing buffer with 2.2% skim milk powder, for 2 h at 37°C. Then, 100 µl of goat anti-rabbit immunoglobulin G labeled with horseradish peroxidase (Sigma-Aldrich) was applied at a dilution of 1∶1000 in washing buffer with 2.2% skim milk powder and incubated for 30 min at 37°C. Finally, citric acid buffer 0.04 M (pH 5.0) in phosphate buffer with 0.07% orthophenylene diamine (Sigma-Aldrich) and 0.22% hydrogen peroxide were added in 100 µl volumes per well. The reaction was halted after 10 min by adding 50 µl of 2.5 M hydrochloric acid. Absorbancies were read at 492 nm on an ELISA reader (Titertek, Helsinki, Finland).

### Challenge/vaccination experiments in bearded dragons (*Pogona vitticeps*)

A total of twenty-two clinically healthy 8-month-old bearded dragons, weighing 80 to 120 g, were used. A first group of five bearded dragons and a second group of six lizards received 200 µl of the incomplete Freund's adjuvant and 100 µl of the Ribi adjuvanted vaccine, respectively. Both vaccines contained 1×10^8^ cfu and were administered through subcutaneous injection at the dorsolateral skin region. Vaccine administration was repeated after 3 weeks. The remaining lizards were injected subcutaneously with saline. A blood sample was collected from each lizard prior to first immunization and subsequently prior to the experimental inoculation. The latter was performed 2 weeks after the booster immunization, by infiltrating the dorsolateral skin of the lizards with a bacterial inoculum in order to induce *D. agamarum* associated dermatitis and/or septicemia. Therefore, the skin of all lizards was infiltrated with 600 µl of a *D. agamarum* suspension containing 3×10^8^ cfu, using a 26 Gauge needle following local disinfection with ethanol as described by Hellebuyck et al. [1], [2]. All lizards were evaluated twice daily for clinical signs related to the development of dermatitis and/or septicemia. Upon development of macroscopic dermatitis, sampling for the presence of *D. agamarum* was performed every 2 days. Lizards showing signs indicative for septicemia, such as anorexia, severe depression and diffuse dark discoloration of the skin, were euthanized out of ethical considerations and necropsy was performed.

### Immunoproteomics

#### Devriesea agamarum *cellysate*

A few colonies of the *D. agamarum* suspension were transferred to 5 ml Luria Broth (LB, Sigma-Aldrich, St. Louis, USA) and incubated at 37°C for 24 h, while shaking. Subsequently, 45 ml LB was added for another incubation of 24 h.

After incubation, the *D. agamarum* bacteria were washed with HBBS and proteins were extracted by means of the ReadyPrep Sequential Extraction Kit (Bio-Rad, Hercules, CA, USA) according to manufacturer's instructions. The extraction buffer was supplemented with tributylphosphine (Biorad), protease inhibitors cocktail (Amersham-GE Healthcare, New Jersey, USA), DNAse and phosphatase inhibitors PP2 and PP3 (Sigma-Aldrich, Steinheim, Germany). Protein quantification was determined by Bradford Coomassie assay.

#### Two-dimensional gelelectrophoresis

One hundred micrograms of lysate were separated in two dimensions as previously described [12]. In short, proteins were solubilized in rehydration buffer (7 M urea, 2 M thiourea, 2% CHAPS, 0.2% carrier ampholytes, 100 mM DTT, bromophenolblue), rehydrated in a Readystrip IPG strip (pI 4–7) (Bio-Rad) and separated according to their iso-electric point in a Protean IEF Cell (Biorad). Subsequently, the strips were incubated in 1.5% DTT and in 4% iodoacetamide and during 10 minutes in equilibrationbuffer (50 mM Tris-HCl pH 8.8, 6 M urea, 20% glycerol, 2% SDS). Separation based on molecular weight was performed on a 10% Tris HCl gel at 150 V for 30 minutes, followed by 200 V for 1 hour. Proteins were visualized with Sypro Ruby staining after fixation in 10% MeOH and 7% acetic acid for at least 30 minutes.

#### Immunodetection

Proteins from the gel were transferred onto a nitrocellulose membrane in a Trans-blot cell filled with 0.1 M CAPS (pH = 11) at 50 V for 30 minutes. Successful transfer was checked with Ponceau S staining. First, the blots were blocked with 0.3% Tween 20 in PBS for minimum 1 hour and then incubated overnight with the primary antibody (pre- and post-immune sera; 1∶100 in blocking buffer). After 3 washing steps (3×5 minutes 0.3% Tw20 in PBS), secondary antibody was applied on the blots for 1 hour (rabbit anti-lizard antibodies, 1∶1000 in blocking solution). Finally, again after 3 washing steps, the blot was incubated with tertiary antibody (goat anti-rabbit HRP-labeled antibodies, 1∶20000 in blocking buffer) followed by chemiluminescence detection.

#### Protein identification

Immunoreactive spots on western blot were matched with their accompanying Sypro stained gel and the immunoreactive proteins were excised from the gel. Peptides were extracted after in gel digestion. Gel pieces were washed twice for 10 minutes in wash solution (25 mM ammonium bicarbonate (ABC); 50% acetonitrile (ACN)), reduced for 10 minutes at 56°C in 100 µL of 10 mM DTT and 25 mM ABC followed by 20 minutes at room temperature. Alkylation was performed with 100 µL 100 mM iodoacetamide and 25 mM ABC for 45 minutes at room temperature. After a washing step, the gel pieces were dehydrated with 100% ACN and modified trypsin (200 ng trypsin in 20 µL ABC, Promega, Madison, USA) was added for overnight digestion at 37°C. Peptides were extracted in two steps: first by means of 50 µL of 50% ACN followed by 100 µL of 100% ACN.

Dried proteins were dissolved in 0.1% formic acid (FA), separated by liquid chromatography as previously described [13]. Mass spectrometric analysis was performed on a ESI Q-TOF Premier (Waters, Milford, MA, USA) in a data dependent mode, where automatically switching between MS (m/z 425–1200) and MS/MS (m/z 50–2100) occurred on up to seven higher charge ions, when the intensity of the individual ions rose above 60 counts per second.

Aminoacid sequences were matched by Mascot Daemon using the in-house sequenced genome from *D. agamarum*. Identified open reading frames (orf) with a p-value of minimum 0.05 were subjected to basic local alignment search tool (BLAST) for alignment with publicly available 35 203 249 non-redundant protein sequences (release 131218), using BLASTx program [14].

The GS De Novo Assembler version 2.6 was used to perform a *de novo* genome assembly using GS FLX reads. The Illumina mate-paired reads were used to scaffold the GS De Novo Assembler contigs using SSPACE Basic 2.0 [15]. The EMBOSS package [16] was used to find orfs with a minimum length of 100 bp in the scaffolds.

### Data analysis

Seroconversion of bearded dragons following vaccination against *D. agamarum* was monitored and compared after administering 5 different vaccines, all containing 1×10^8^ CFU, but each assembled with a specific adjuvans. A total of 5 vaccines (5 different adjuvantia) were evaluated by immunization of 5 lizards in each experimental group. A lizard was considered to have seroconverted if the OD after immunization reached at least the mean OD value derived from repeated analyses of individual preimmune serum samples plus 3 times the standard deviation. By this means the number of seroconverted lizards in each group could be identified (with a maximum of 5 seroconverted animals).

Due to the limited numbers of animals in each sample group (5 animals in each group), the Fisher's Exact test was applied to screen for possible statistical differences between the use of the different vaccines (i.e. different adjuvantia). The same test was applied to monitor the effectiveness of the 2 most suitable *D. agamarum* vaccines in a challenge/vaccination experiment with 22 lizards.

A statistical difference between groups was assumed if p<0.05 (SPSS statistics version 22.0, IBM, Belgium).

## Results

### Immunization of bearded dragons (*Pogona vitticeps*) against *Devriesea agamarum* results in seroconversion

#### ELISA results

The optical density (OD) values of lizard preimmune serum samples showed overall high inter-individual variability. Accordingly, cut-off OD values were calculated for each lizard individually in all experiments as the mean OD value derived from repeated analyses of individual preimmune serum samples plus 3 times the standard deviation. A lizard was considered to have seroconverted when OD values higher than this cut-off OD were demonstrated for sera collected after immunization. OD values were determined in 3-fold for all serum samples obtained from immunized animals.

**Figure 1:**
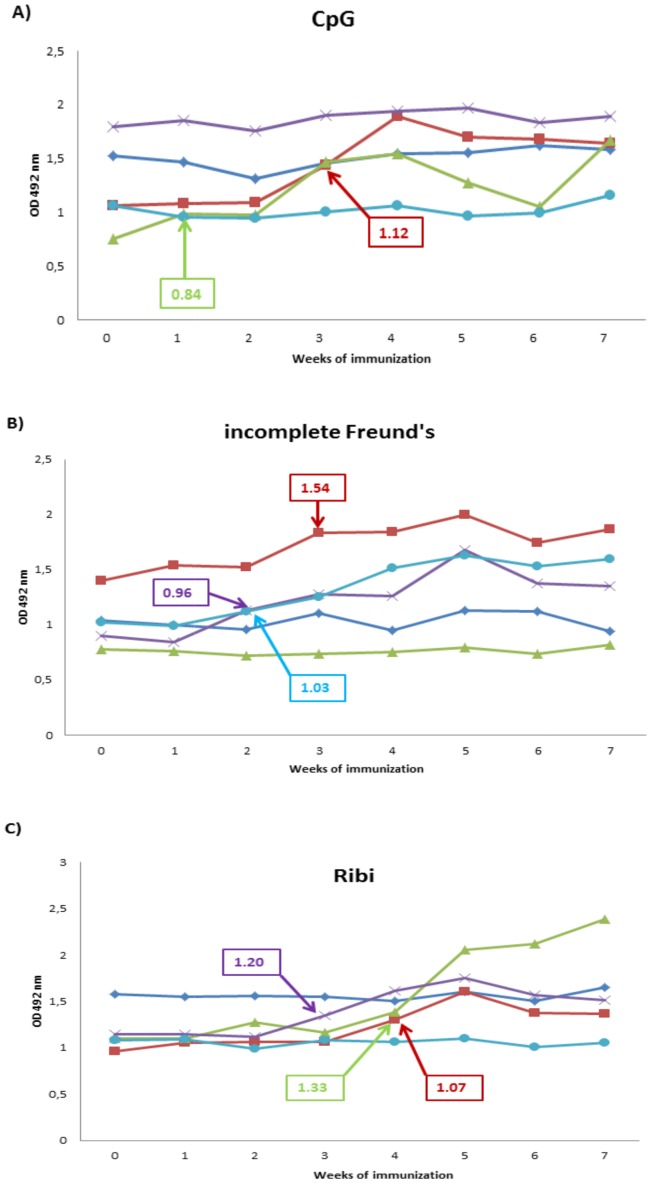
Antibody response of bearded dragons (*Pogona vitticeps*) following immunization against *Devriesea agamarum*. Results of ELISA of immune and preimmune (0 weeks) lizard sera at 1∶64 dilution following first immunization at week 0 and booster immunization at week 3 with a formalin-inactivated *Devriesea agamarum* vaccine containing (A) CpG adjuvant, (B) incomplete Freund's adjuvant and (C) Ribi adjuvant. Each vaccine formulation was administered to 5 lizards and serum was collected weekly during a 7-week period. A cut-off value was calculated for each lizard individually as the mean OD value derived from repeated analyses of individual preimmune serum samples plus 3 times the standard deviation. The moment of seroconversion, defined as the point in time when the demonstrated OD values exceed the cut-off value, and the corresponding cut-off value is indicated for the individual lizards (arrow).

During the first experiment, conducted to evaluate the serological response of bearded dragons after immunization with 5 different *D. agamarum* vacines, seroconversion was observed in 2 out of 5 lizards in the groups that were administrated the CpG vaccine and in 3 out of 5 lizards that were immunized with the Ribi or incomplete Freund's vaccine. All showed seroconversion prior to booster vaccination except for 2 out of 3 lizards, immunized with the Ribi adjuvanted vaccine, which seroconverted 1 week after booster vaccination (Figure 1). In the groups that were vaccinated with the aluminium hydroxide and curdlan vaccine, seroconversion could not be demonstrated in any of the lizards. No significant difference in the number of seroconverted lizards could be observed for the 5 different *D. agamarum* vaccines (Fisher's Exact test, p>0.05). A remarkable trend, however, was observed for both the Ribi and incomplete Freund's vaccine, indicating a more successful immunization when compared to the effect of immunization with the aluminium hydroxide and curdlan vaccine (Fisher's Exact test; p = 0.083).

Following immunization of bearded dragons with the incomplete Freund's and Ribi adjuvanted vaccine during the challenge/vaccination experiment, seroconversion was observed in 1 out of 5 and 2 out of 6 immunized lizards respectively, 5 weeks after primo-vaccination. In none of the sham immunized lizards could seroconversion be demonstrated. These results could not be proven to be significantly better to the sham immunization of lizards in which no seroconversion could be observed (Fisher's Exact test).

#### Adverse effects following immunization and challenge

Except for the lizards that received the Ribi adjuvanted vaccine, localized adverse immunization effects were observed following vaccine administration in all other groups, consisting of extensive granulomatous inflammation at the immunization sites (Table 1). Immunization did not seem to elicit generalized adverse effects in any of the lizards.

**Table 1:** Localized adverse effects in bearded dragons following subcutaneous immunization with various formalin-inactivated *Devriesea agamarum* vaccines.

Vaccine	Adverse effects	Number of lizards with adverse effects (n = 5)		Onset (average time in days following first immunization)		Maximum size of nodule (average, cm^3^)	
		[1]	[2]	[1]	[2]	[1]	[2]
**CpG**	Yes	2	3	13	50	1	1
**Incomplete Freund's**	Yes	5	5	1	21	1.5	1.5
**Ribi**	No	-	-	-	-	-	-
**Aluminium hydroxide**	Yes	5	5	9	30	0.75	0.75
**Curdlan**	Yes	0	4	-	40	-	0.4

[1], [2]: first immunization and second immunization (day 21) respectively.

Following primo-vaccination with the CpG vaccine, large subcutaneous nodules with an average size of 1 cm diameter were observed at day 13 in two lizards. The size of these nodules gradually decreased to 0.5 cm diameter average at day 70 and remained constant in size during the entire observation period. Following booster inoculation 3 bearded dragons developed a subcutaneous nodule of 1 cm diameter at the inoculation site at day 50. These nodules remained present throughout the entire observation period. In all lizards that received the incomplete Freund's vaccine during the first experiment, conducted to compare the effect of 5 different vaccines, large subcutaneous nodules of 1.5 cm diameter on average were observed immediately following first immunization. Following second immunization similar nodular lesions developed in all 5 lizards. In two lizards the nodules, induced following second immunization decreased gradually in size from day 60 and disappeared almost completely at the end of the observation period. All other nodules remained present and did not decrease in size during the entire observation period.

Immediately following administration of the incomplete Freund's vaccine during the challenge/vaccination trial, demarcated nodular lesions of 0.8 cm diameter on average were observed in all lizards. After the second immunization identical adverse effects were observed. Although an initial decrease in size to 0.6 cm on average was noted at day 5, the size of the nodular lesions remained constant during the rest of the trial.

In the group of lizards that was vaccinated with aluminium hydroxide all animals developed subcutaneous nodules with an average diameter of 0.75 cm at day 5 to 13, which resolved in two lizards at day 35 on average and started decreasing in size from day 50 in the remaining lizards. Following second immunization identical adverse effects were seen in all lizards at day 30. At the end of the observation period, all lesions in every lizard had disappeared except for one lizard.

None of the lizards belonging to the curdlan vaccine group showed local or generalized adverse effects following first immunization. After second immunization, however, relatively small nodules were noted in 4 lizards at day 40 with an average diameter of 0.4 cm. In 4 out of 5 lizards, these lesions almost completely resolved. In one lizard a small subcutaneous nodule could be observed until the end of the observation period.

At the end of the observation period of the first experiment, full thickness biopsies of the subcutaneous nodules were collected in two lizards that were immunized with the incomplete Freund's adjuvanted vaccine using a 3 mm diameter biopsy punch (Paramount Surgimed Ltd., New Dehli, India). Tissues were fixed in 10% neutral buffered formalin, processed routinely, embedded in paraffin and stained with hematoxylin and eosin. Histological sections revealed multifocal subcutaneous granuloma's of variable size containing few cells. The granuloma's consisted of a fibrous capsule surrounding different layers of macrophages and a central eosinophilic core. In addition, dermal infiltration of lymphocytes, plasma cells, heterophils and macrophages was observed. The presence of bacteria within the cytoplasm of the latter macrophages and in the core of the granuloma's was confirmed by periodic acid Shiff staining.

### Seroconversion following autovaccination against *Devriesea agamarum* confers protection against the development of septicemia but not dermatitis

During the challenge/vaccination experiment, the vaccinated as well as the non-vaccinated lizards developed dermatitis in the inoculated region of dorsolateral skin at 5 days on average post inoculation. The dermal lesions evolved to encrusted, discolored areas of infected skin with purulent discharge.

In the incomplete Freund's vaccinated group, none of the vaccinated animals showed obvious clinical signs indicative for septicemia. One of these lizards, however, showed a 3 day period of anorexia from the 9^th^ until the 11^th^ day post inoculation. In the Ribi immunized group, 3 lizards showed anorexia from 6 days post inoculation until the 9^th^ day on average post inoculation. From then on, the latter bearded dragons seemed fully recovered and remained in a general good condition throughout the trial.

Eight non-vaccinated lizards showed decreased appetite and demonstrated other signs suggestive for systemic disease at the 4^th^ day on average post inoculation. These clinical signs became progressively worse and consisted of anorexia, pronounced apathy, diffuse dark discoloration of the skin and intermittent but severe dyspnea. Five of the latter lizards reached ethical endpoints and were humanely euthanized at day 9, 10, 12, 13 and 21 post inoculation respectively. The general condition of the 3 other lizards that displayed signs of septicemia gradually improved. These animals regained appetite and seemed fully recovered at day 15 on average post inoculation.

From all lizards *D. agamarum* could be isolated from the inoculated areas of skin until the end of the trial. Following necropsy of the 5 euthanized bearded dragons, *D. agamarum* was isolated in pure and abundant culture from skin, liver, spleen and kidney. In 3 of the latter lizards, *D. agamarum* was additionally cultured from the bone marrow.

### Antigen identification of Ribi vaccine

**Figure 2:**
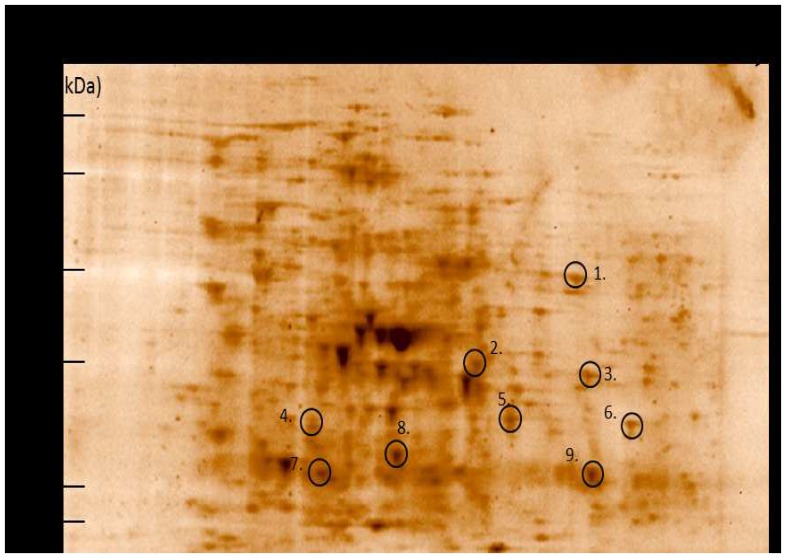
Sypro-staining of a two-dimensional gel of *Devriesea agamarum* cellysate. Immunoreactive spots that emerge after vaccination in at least 2 of the 3 animals (3.2, 3.3, 3.4) were identified by mass spectrometry. Identification of the marked spots are depicted in table 2.

Sera collected 5 weeks after primo vaccination from the three lizards that showed seroconversion after Ribi vaccination were used for immunoblotting experiments (referred to as 3.2, 3.3 and 3.4). Therefore, for each animal 2 western blots with *D. agamarum* cell lysates were made, one was incubated with serum before vaccination and the other with serum after vaccination. Both western blots were compared to each other. Differential immunoreactive spots on the post-vaccination blot were matched with their accompanying gel and identified by means of mass spectrometry. Immunoreactive spots that were present in at least 2 of the 3 animals and which yielded, after excision from their accompanying gel, the same protein identifications are summarized in figure 2 and table 2.

**Table 2:** Protein identifications with a p-value <0.05 of immunoreactive spots from Figure 2.

	N° serum	Mass	Score	Matches	Protein name
**1**	**3.3**	53531	1022	20	Dihydrolipoamide dehydrogenase
	**3.4**	53531	1205	26	Dihydrolipoamide dehydrogenase
**2**	**3.3**	40579	148	5	serine/threonine protein kinase
	**3.4**	40579	345	7	serine/threonine protein kinase
**3**	**3.3**	36909	171	4	dTDP-glucose 4,6-dehydratase
	**3.4**	36909	128	3	dTDP-glucose 4,6-dehydratase
**4**	**3.3**	33555	228	5	Cysteine synthase
	**3.4**	33555	98	2	Cysteine synthase
**5**	**3.3**	40579	92	4	serine/threonine protein kinase
	**3.4**	40579	118	4	serine/threonine protein kinase
**6**	**3.3**	31255	521	10	NAD-dependent protein deacetylase, SIR2 family
	**3.4**	31255	632	14	NAD-dependent protein deacetylase, SIR2 family
**7**	**3.3**	27611	588	9	Triosephosphate isomerase
		19111	254	4	Translation elongation factor Tu
		37705	184	3	Fructose-bisphosphate aldolase
	**3.4**	27611	733	13	Triosephosphate isomerase
		43870	315	5	Translation elongation factor 1A (EF-1A/EF-Tu)
		37705	160	3	Fructose-bisphosphate aldolase
**8**	**3.2**	30902	479	10	Putative oxidoreductase
		37705	123	2	Fructose-bisphosphate aldolase
	**3.3**	30902	186	5	Aldo/keto reductase, diketogulonate reductase
		37705	139	3	Fructose-bisphosphate aldolase
	**3.4**	37705	366	7	Fructose-bisphosphate aldolase
**9**	**3.3**	32098	398	6	ATP synthase, F1 delta subunit
	**3.4**	32098	640	9	ATP synthase, F1 delta subunit

N° serum: the animal from which this immunoreactive spot was identified; mass: molecular weight of the identified protein; score: score of protein identification determined by Mascot Daemon; matches: number of peptides identified per open reading frame (orf); protein name: name of the protein after blasting the identified orf.

Serum from animal 3.3 and 3.4 showed reactivity against dihydrolipoamide dehydrogenase, serine/threonine protein kinase, dTDP-glucose 4,6-dehydratase, cysteine synthase, triosephosphate isomerase, translation elongation factor Tu, and ATP synthase, F1 delta subunit. Aldo/keto reductase was identified as immunoreactive in serum 3.2 and 3.3. Two proteins, fructose-bisphosphate aldolase and aldo-keto reductase, were found as immunoreactive spot in all 3 animals.

## Discussion

Entry control and quarantine of newly acquired lizards, the use of appropriate disinfection procedures and antimicrobial treatment have previously been described as essential aspects of an integrated approach to prevent and eliminate devrieseasis from captive lizard collections [2], [4], [5], [6]. In collections with persistent and high mortality associated to *D. agamarum* infection, autovaccination could serve as an additional powerful and even indispensable tool towards disease control.

Although generally considered as the serodiagnostic method of choice, indirect ELISAs require the availability of specific antispecies monoclonal or polyclonal antibodies [17]. Commercial antibodies for use in serological testing of reptiles are largely unavailable forcing investigators wishing to work with these types of reagents to produce their own [17]. The indirect ELISA used in this study was not developed to design a practical serodiagnostic test to determine exposure of lizards to *D. agamarum*, but it did allow the unambiguous detection of seroconversion in lizards after immunization against *D. agamarum*. The development of specific rabbit anti-lizard monoclonal or polyclonal serum after immunization of rabbits with purified *P. vitticeps* immunoglobulins could be an important step to enhance and broaden the applicability of the used indirect ELISA.

Based on the overall results of this study, there was no evidence for affinity maturation following the use of the described vaccination protocols against *D. agamarum* in bearded dragons. Booster injections of the antigen or experimental inoculation did not result in an obvious increase of OD values over a longer period of time. This finding corresponds with those observed following immunization of several lizard species with a variety of antigens [18], [19]. An obvious increase of the affinity of low-molecular-weight antibodies on the other hand was previously demonstrated in immunized tortoises, several snake species, alligators and desert iguana's (*Dipsosaurus dorsalis*) [18], [20], [21]. In one lizard that received the Ribi adjuvanted vaccine however, it should be noted that a steady increase in serum OD values was observed with an additional rise following booster immunization to reach a peak value 7 weeks following primo-vaccination.

In order to induce a strong antibody response adjuvants are routinely used as nonspecific stimulators of the immune response [22]. Incomplete Freund's and Ribi adjuvant have been previously used in reptile immunization studies and proved to evoke a strong and prolonged immune response [17], [22], [23]. The use of incomplete Freund's adjuvant, however, may result in considerable adverse effects, mostly presenting as granulomatous inflammatory responses and focal necrosis [22], [24]. During this study these adverse effects were highly prominent in incomplete Freund's vaccinated lizards. In contrast, the newer synthetic adjuvant Ribi did not elicit adverse effects and induced overall comparable levels of seroconversion as the incomplete Freund's adjuvanted vaccine. For this reason the proteomics research was focused on serum obtained from Ribi vaccinated animals.

The development of a cell mediated immune response following the use of the different vaccine formulations against *D. agamarum* was not investigated during this study. Antigen specific cell mediated immune responses have been detected in different reptile species [7], [23] and cell mediated immunity may contribute to the partial protection following immunization against *D. agamarum* infection observed in this study. To assess the overall immune responsiveness in bearded dragons as a result of immunization against *D. agamarum*, evaluating the cell mediated immune and correlating the latter response with the antibody response would be essential.

As the described immunization with incomplete Freund's and Ribi vaccine conferred partial protection against *D. agamarum* associated disease in lizards, variation in antigen composition or mode of antigen inactivation, route of administration and booster interval and frequency should be strongly considered and may result in a more favorable outcome towards the development of an immunization protocol aiming to prevent *D. agamarum* induced dermatitis in lizards.

Proteomic analysis yielded two *D. agamarum* antigens that may be interesting candidates for vaccine development, fructose-bisphosphate aldolase and aldo-keto reductase. Fructose-bisphophate aldolase is a zinc-binding reversible enzyme in the glycolysis. It catalyzes the cleavage of fructose-1,6-bisphosphate to dihydroxyacetone phosphate and D-glyceraldehyde-3-phosphate [25]. Aldo-keto reductase represents a superfamily of soluble NAD(P)(H) oxidoreductases whose chief purpose is to reduce aldehydes and ketones to primary and secondary alcohols [26]. However, the protein names are based on blasting since no annotated sequence database is available for *D. agamarum*. Proteins that are unique to this bacterium will therefore be missed. The latter seemed not the case since after blasting the identified proteins were all found with high alignment scores in *Brachybacterium* species as well, a species closely related to *D. agamarum* from which sequenced genes were already annotated [27].

One could wonder whether cytosolic proteins can be involved in establishing an immune response. Several reports, however, have already stated the transient presence of cytosolic proteins at the cell surface even without the presence of a signal peptide [28], [29]. Accordingly, fructose-bisphophate aldolase has already been detected at the cell surface of *Streptococcus pneumoniae* bacteria and was found to be a novel *S. pneumoniae* vaccine candidate, illustrating that proteins which are considered as cytosolic can be immunogenic [29].

## Conclusions

In summary, the use of formalin-inactivated *D. agamarum* Ribi adjuvanted as well as incomplete Freund's adjuvanted vaccines result in seroconversion in lizards and confer partial protection against *D. agamarum* associated disease. The latter vaccine however, provokes the development of persistent granulomas following subcutaneous administration. Proteome analysis of the antigens that bind with sera from animals with seroconversion after Ribi vaccination reveals several possible vaccine candidates such as fructose-bisphosphate aldolase and aldo-keto reductase.
